# The Potential of Polyphenols in Modulating the Cellular Senescence Process: Implications and Mechanism of Action

**DOI:** 10.3390/ph18020138

**Published:** 2025-01-22

**Authors:** Larissa Della Vedova, Giovanna Baron, Paolo Morazzoni, Giancarlo Aldini, Francesca Gado

**Affiliations:** 1 Department of Pharmaceutical Sciences, University of Milan, Via Mangiagalli 25, 20133 Milan, Italy; larissa.dellavedova@unimi.it (L.D.V.); giovanna.baron@unimi.it (G.B.); giancarlo.aldini@unimi.it (G.A.); 2 Divisione Nutraceutica, Distillerie Umberto Bonollo S.p.A, Via G. Galilei 6, 35035 Mestrino, Italy; paolo.morazzoni@bonollo.it

**Keywords:** polyphenols, senolytic, senomorphic, cellular senescence

## Abstract

**Background**: Cellular senescence is a biological process with a dual role in organismal health. While transient senescence supports tissue repair and acts as a tumor-suppressive mechanism, the chronic accumulation of senescent cells contributes to aging and the progression of age-related diseases. Senotherapeutics, including senolytics, which selectively eliminate senescent cells, and senomorphics, which modulate the senescence-associated secretory phenotype (SASP), have emerged as promising strategies for managing age-related pathologies. Among these, polyphenols, a diverse group of plant-derived bioactive compounds, have gained attention for their potential to modulate cellular senescence. **Methods**: This review synthesizes evidence from in vitro, in vivo, and clinical studies on the senolytic and senomorphic activities of bioactive polyphenols, including resveratrol, kaempferol, apigenin, and fisetin. The analysis focuses on their molecular mechanisms of action and their impact on fundamental aging-related pathways. **Results**: Polyphenols exhibit therapeutic versatility by activating SIRT1, inhibiting NF-κB, and modulating autophagy. These compounds demonstrate a dual role, promoting the survival of healthy cells while inducing apoptosis in senescent cells. Preclinical evidence indicates their capacity to reduce SASP-associated inflammation, restore tissue homeostasis, and attenuate cellular senescence in various models of aging. **Conclusions**: Polyphenols represent a promising class of senotherapeutics for mitigating age-related diseases and promoting healthy lifespan extension. Further research should focus on clinical validation and the long-term effects of these compounds, paving the way for their development as therapeutic agents in geriatric medicine.

## 1. Introduction

Senescence and aging are closely related but not synonymous. Aging is the overall process of becoming older, while senescence focuses on the specific cellular changes which contribute to the aging process. In more detail, aging is the physiological process of a time-dependent functional decline, a predictable, natural, gradual process of an organism which occurs due to pro-aging mechanisms such as DNA damage, lipid-peroxidation, and protein misfolding, resulting in cell death or senescence. Cellular senescence was first identified in fibroblasts by Hayflick and Moorhead when cultured primary cells were found to undergo a limited number of cell divisions in vitro [1,2], but this was considered an in vitro artifact [3] rather than an aging hallmark which may potentially serve as a connecting factor among other known hallmarks of aging [4]. Nowadays, senescence is known as a mechanism of fundamental aging and cellular fate in eukaryotic life, following intrinsic or extrinsic factors, including oncogene activation, oxidative stress, mitochondrial dysfunction, irradiation, and exposure to chemotherapeutics [5].

**Figure 1:**
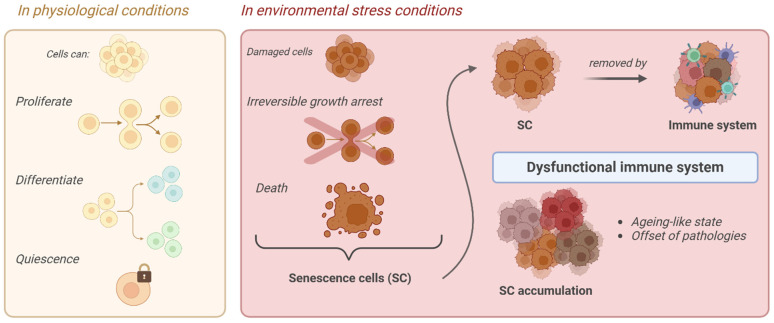
Schematic representation of senescence cell (SC) formation.

Regarding oxidative stress, it has been hypothesized that during aging, cells lose their functions because of ROS-induced damage [6]. In physiological conditions, cells undergo proliferation, quiescence, or differentiation, but in the presence of environmental stress, severely damaged cells undergo stable and irreversible growth arrest, losing their capacity to divide, exiting cell cycle, and entering a state of cellular senescence or cell death. These cells are then referred to as senescence cells (SCs) (Figure 1) [7,8,9,10,11,12].

Notably, this process is not necessarily undesirable, since SCs are important mediators of various physiological mechanisms such as tumor development and aging. During early tumor initiation, cellular senescence (CS) acts as a barrier by inducing cell cycle arrest and preventing malignancy. This process is accompanied by a shift in cellular metabolism, characterized by enhanced mitochondrial pyruvate dehydrogenase activity and increased mitochondrial ROS levels. CS also triggers the senescence-associated secretory phenotype (SASP), which has both tumor-suppressive and pro-tumorigenic roles. The SASP can recruit immune cells, promote immune surveillance, and aid fibrosis resolution by attracting macrophages. However, in some contexts, SASP components like IL-6 and IL-8 can support tumor growth by driving epithelial-mesenchymal transition (EMT) and cancer stem cell phenotypes, as well as by promoting angiogenesis and invasion through the secretion of VEGF and matrix metalloproteinases (MMPs). The effect of the SASP on tumor progression depends on the stage of tumor development, with a weakened immune response in later stages allowing SASP factors to enhance tumor growth [7,8].

To better understand the physiological roles of senescence, animal models have been developed to visualize and eliminate senescent cells in vivo. Studies have shown that senescent fibroblasts and endothelial cells appear early during wound healing, where they contribute to tissue repair by secreting platelet-derived growth factor AA (PDGF-AA), accelerating wound closure [9]. Additionally, developmentally programmed senescence occurs during embryonic development in the mesonephros and endolymphatic sac, where it is regulated by p21 and plays a key role in tissue remodeling, representing the evolutionary origin of damage-induced senescence [10].

In aged tissues, senescence is marked by the increased expression of p16 and p21, which are commonly used as markers for identifying senescent cells [11]. A study by Yousefzadeh et al. [12] examined senescence in both wild-type (WT) and progeroid Ercc1−/Δ mice, which have accelerated aging due to a DNA repair deficiency. In both groups, the expression of senescence markers was higher in aged mice compared with the young controls, with varying levels across different tissues. Specifically, elevated levels of SASP markers, including IL-6, are correlated with the progression of age-related pathologies.

Current research in this domain is now focused on understanding the effect of bioactive molecules as modulators of cellular senescence, and among these polyphenols are a promising class of compounds. Polyphenols are phytocompounds found in fruits and vegetables and nowadays are considered important constituents of a healthy diet due to their well-documented role in many diseases and, more specifically, in the prevention and treatment of oxidative damage and inflammation [13]. Polyphenols are represented by several principal groups according to the number of phenolic rings and the structural elements of those rings, namely flavanols (catechins, epicatechin, and procyanidins), flavonols (quercetin glycosides), stilbenes, phenolic acids (chlorogenic, gallic and coumaric acids), dihydrochalcones (phloretin glycosides), and anthocyanins (cyanidin glycosides) [14]. Studies reported in the literature have already highlighted the signaling pathways modulated by polyphenols in order to exert their beneficial effects, such as nuclear factor erythroid 2-related factor 2 (Nrf2), nuclear factor-κB (NF-κB), mammalian target of rapamycin (mTOR), and sirtuins, as well as key processes such as autophagy, immunomodulation, cell proliferation, and apoptosis [15]. Moreover, evidence suggests that long-term consumption of dietary polyphenols confers a protective role in a multitude of age-related degenerative diseases and even organismal longevity [16].

This review aims to give an overview of the studies reported in the recent literature concerning the therapeutic potential and mechanism of action of the major polyphenols in the senescence process, highlighting the importance of these natural compounds in being considered as possible future nutraceuticals.

## 2. Senescence Cells (SCs)

SCs are physiologically removed by the immune system. However, in the presence of an increasingly dysfunctional immune system in elderly individuals or “do not eat me” signals carried by some of them, in both cases, they may manage to evade their clearance. In this way, their accumulation begins with the consequences of an accelerated aging-like state and the onset of different pathologies [17]. SCs display distinctive features. They already exhibit a flattened morphology and an increased cell size in the early stages of senescence, initiated as a consequence of one or more of the aforementioned stimuli. At this point, SCs also present a remodeling of nuclear chromatin, the loss of lamin-B1, the induction of an oxidative metabolism in mitochondria, and activation of the SC’ anti-apoptotic pathway (SCAP), making SCs resistant to death. Moreover, senescence-associated β-galactosidase activity (SA-β-gal) caused by an increase in lysosomal activity is also detected. In addition, SCs are accompanied by a senescence-associated secretory phenotype (SASP), namely a chronic pro-inflammatory behavior which mediates the secretion of numerous pro-inflammatory cytokines, proteases, growth factors, and other extracellular proteins [18,19,20].

**Figure 2:**
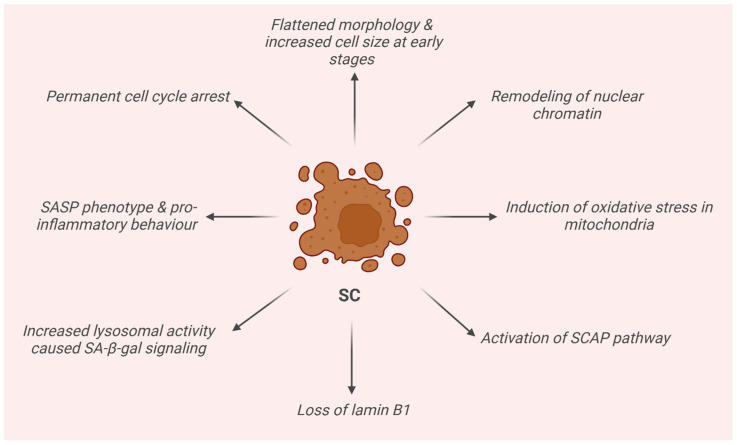
Schematic representation of the main features of senescence cells (SCs).

Another fundamental characteristic is permanent cell cycle arrest. In fact, SCs cannot reenter the cell cycle through any known physiological stimulation, and some of them contain senescence-associated heterochromatin foci (SAHF), which helps establish and maintain cell cycle arrest by suppressing proliferation-related gene expression [21]. Notably, SCs remain highly active metabolically and are able to modulate the surrounding microenvironment [18,19]. Furthermore, these cells are characterized by shortened telomeres, hypertrophy, an altered chromatin structure, accumulation of DNA damage and reactive oxygen species (ROS), and the development of senescence-associated heterochromatic foci (SAHFs) [22]. Also, autophagy is thought to be tightly linked to senescence, even if the precise connection is still unknown. Autophagy contributes to maintaining homeostasis in an organism through the degradation of damaged macromolecules or organelles through lysosomes in cells and may be associated with both senescence and apoptosis [23]. Indeed, increased autophagy leads to cell death, but its inhibition can lead to senescence. Recent studies confirmed that the outcome of the interplay between autophagy and senescence depends on the cell type, microenvironment, and probably other factors which are still unclear [23,24]. The activation of cell cycle p53, p16Ink4a, and p21CIP1 inhibitory pathways have been identified and recently reported in the literature as senescence-associated, being triggered by internal or external stimuli and DNA damage response (DDR). The first phase, where senescence is still reversible and in which cells stop dividing but are metabolically active, is controlled by p53 and p21, the latter of which is the main p53 effector and involved in G1/S and G1/M cell cycle arrest, modulating the activity of p53 targets CDC25B and CDC25C in particular and survival (Figure 2) [25]. In the second phase, senescence becomes irreversible, and p16 plays the major role. In particular, it indirectly modulates the Rb family members and E2F target gene expression. Senescence progression is strictly correlated to E2F-4, which regulates E2F target genes, and the complex Rbl2/p130, which is implicated in cell cycle arrest and senescence maintenance [26,27,28,29]. In actuality, even the second phase is not to be considered irreversible anymore. Indeed, recent studies demonstrated that in some cases, SCs can reenter the cell cycle in tumors [30] or be reprogrammed into pluripotent stem cells [31].

## 3. Therapeutic Approaches

The elimination of SCs from cell cultures, tissues, and even whole organisms represents a promising therapeutic strategy to prevent the onset of chronic diseases and extend healthy lifespans, and it may be possible with the assistance of senolytic drugs [32]. Senolytic drugs are developed to target a specific cell type (SCs) and are a step forward from the usual one target, one drug, one disease approach since it is not a signaling pathway, receptor, or enzyme they address their action toward. Indeed, in order to define if a drug may be a senolytic, a modified set of Koch’s postulates has been suggested [33]:

1. The SCs and phenotype must be strictly associated, and if an individual does not present SCs, then he or she will not present the phenotype;
2. The phenotype is caused by an induced accumulation of SCs, and as a consequence, removing the induced SCs ameliorates the phenotype;
3. Senolytics have minimal effects on the phenotype in young individuals without SCs;
4. An intermittent administration of senolytics has been proven to be effective;
5. Senolytic drugs are able to alleviate various age-related diseases.

Moreover, it is worth mentioning that after senolytics clear SCs, these cells take some time to accumulate again, and this “hit and run” regimen allows reducing any potential adverse effects which senolytic drugs may present. As senolytic drugs represent a novel therapeutic approach aimed at targeting the underlying processes of aging to prevent or alleviate age-related conditions, multiple diseases, geriatric syndromes, and declines in physical resilience, it is crucial to carefully assess the risk-benefit balance in early clinical trials. The potential short- and long-term side effects of clearing senescent cells remain uncertain [33]. Senolytic drugs have been developed according to different approaches. Initially, many current senolytic drug candidates target anti-apoptotic pathways involving the BCL-2 protein family, p53, and the PI3K/AKT axis. This strategy derived from the assumption that the majority of SCs present a pro-apoptotic, tissue-destructive SASP and are resistant to apoptosis, meaning they depend on anti-apoptotic, pro-survival pathways to avoid death [34,35,36]. A second generation of senolytic drugs has been identified thanks to computational studies based on an increase in lysosomal mass and senescence-associated β-galactosidase activity, as well as targeting the modulation of immune clearance of SCs [37,38,39]. Another approach concerns the use of SASP inhibitors, known also as senomorphic drugs. They directly or indirectly suppress the SASP without eliminating the SCs by inhibiting NF-κB, the janus kinase/signal transducer and activator of transcription (JAK-STAT) pathway, the serine/threonine protein kinase mTOR, mitochondrial complex-1-related or 4-related targets, or other pathways involved in the induction and maintenance of the SASP [40,41,42,43]. When comparing senolytic drugs to SASP inhibitors, there are important differences to consider. Firstly, SASP inhibitors need continuous treatment to suppress the SASP, while senolytics present intermittent “hit and run” administration which reduces the side effects that normally manifest after a week of continuous treatment. The continuous administration of SASP inhibitors may lead also to off-target effects due to the suppression of cytokine secretion, such as nephrotoxicity, metabolic impairment, and susceptibility to infections [44,45,46]. Moreover, an interesting hypothesis suggests that senolytic-induced apoptosis of destructive SASP-expressing senescent cells, which are concentrated at sites of pathology, might contribute to the beneficial effects of senolytics [47]. In general, the elimination of all senescent cells or inhibition of the SASP might be detrimental where SCs are beneficial, but in any case, targeting the persisting, tissue-destructive SASP-expressing SCs might have superior therapeutic potential and fewer off-target effects [48].

## 4. Anti-Cellular Senescence Activity by Isolated Dietary Polyphenols

### 4.1. Senomorphic Activity

#### 4.1.1. Resveratrol

Resveratrol (3,5,4′-trihydroxystilbene) is a stilbenoid polyphenol present in many fruits and vegetables and especially red wine. Its activity in cellular senescence relies on its ability to activate silencing information regulator 2-related enzyme 1 (SIRT1), which is an NAD+-dependent deacetylase involved in many signaling and transcriptional pathways implicated in cellular senescence and aging. Indeed, this enzyme physiologically decreases with age, but it was demonstrated that an increase in its expression leads to a reduction in cellular senescence and extended lifespans in many organisms [49]. It is interesting to note that this natural compound presents a biphasic effect on cellular senescence, depending on its concentration. At low concentrations (generally below 10 µM), resveratrol exhibits senomorphic or antioxidant behavior, preventing cellular senescence and suppressing SASPs. In particular, it belongs to the first senomorphics which were mainly discovered through serendipity, together with rapamycin, metformin, and aspirin [50]. An in vitro study conducted by Xia et al. on endothelial progenitor cells (EPCs) demonstrated that resveratrol was able to delay the onset of EPC senescence, and this effect was accompanied by activation of telomerase through the PI3K-Akt signaling pathway [51]. In vascular smooth muscle cells from aged rhesus monkeys, 1 µM of resveratrol attenuated mitochondrial O(2)(−) production and suppressed SASP factors by inhibiting NF-kB and upregulating Nrf2 pathways [52]. In 2010, resveratrol was tested in vitro in a normal human fibroblast cell line derived from fetal lung tissue (MRC5), with 5 µM inducing a small increase in the total number of replications completed by the cultures at senescence, showing protective effects against DNA oxidative damage and reducing senescence-associated increases in nuclear size and DNA content. Moreover, diminished levels of acetylated forms of the H3 and H4 histones and p53 protein were also detected [53]. Resveratrol was also tested in an in vivo model of male mice on a high-fat diet and was shown to produce changes associated with longer lifespans, including increased insulin sensitivity, reduced insulin-like growth factor-1 (IGF-I) levels, increased AMP-activated protein kinase (AMPK) and peroxisome proliferator-activated receptor-γ coactivator 1α (PGC-1α) activity, an increased mitochondrial number, and improved motor function. Interestingly, it was only able to extend the lifespans of male mice on high-fat diets and not those on standard diets, chow diet-fed C57/B6 mice, or heterozygous mice, demonstrating improved health and prevention of early mortality associated with obesity [54,55,56]. More recently, resveratrol was tested in vitro on two different cell models, namely microglia BV2 cells and rat pheochromocytoma cells (PC12 cells), to explore its effect on oxysterol 27-hydroxycholesterol-induced neural senescence. Resveratrol, as an SIRT1 agonist, was able to attenuate 27-hydroxycholesterol-induced neural senescence in both cell lines by inhibiting signal transducer and activator of transcription 3 (STAT3) signaling via SIRT1 [57]. In the same study, it was also tested in vivo using a zebrafish model, showing a decrease in 27-hydroxycholesterol-induced locomotor behavior disorders and aging in the spinal cords of zebrafish larvae, which was still associated with the activation of SIRT1-mediated STAT3 signaling. It was also shown at 1 µM to inhibit cellular senescence of human bone marrow stromal stem cells by reducing the levels of SASP, gene markers associated with senescence (p53, p16, and p21), and intracellular ROS and increasing gene expression of the enzymes protecting cells from oxidative damage (HMOX1 and SOD3) [58]. Instead, when we consider higher concentrations (typically 25 µM), resveratrol shows a pro-oxidant behavior, triggering growth arrest and the induction of senescence or apoptotic death, as confirmed by studies conducted on multiple cell lines [59]. Therefore, it is evident that aside from the concentrations used, the divergent or opposing effects of resveratrol on cellular senescence and other biological processes may be due to cell and tissue types, the timing and routes of dosing, sex, genetic background, and diet compositions (Table 1) [50].

#### 4.1.2. Kaempferol

Kaempferol (KAE) is a natural flavonol belonging to the class of flavonoids, being present in different plant species and known for its anti-inflammatory, antioxidant, and anticancer properties. In a study by Lim et al. in 2015, KAE was demonstrated to strongly inhibit the expression of SASPs via the inhibition of NF-κB, p65 activity through the interleukin-1 receptor-associated kinase (IRAK1) IκBα signaling pathway, and especially the expression of IκBζ. The research was conducted in vitro on bleomycin-induced senescent BJ fibroblast, and KAE yielded the most promising results, together with apigenin, in a total of five initially tested naturally occurring flavonoids [60]. A few years later, KAE was found to be able to alleviate the aging of porcine oocytes through a reduction of oxidative stress and improved mitochondrial function [61]. Recently, a KAE derivative, KAE tetrasaccharides, was demonstrated to be able to significantly reduce cellular senescence and increase collagen fiber in skin cells and human skin equivalent. In multiple participants in one study, a clinical enhancement in skin appearance was detected, together with an improvement in the histological appearance of skin tissue and the extracellular matrix, which was supported by immunohistochemical studies. These results were achieved following pyruvate dehydrogenase kinase 1 (PDK1) inhibition [62]. One of the last studies concerning KAE was conducted in nucleus pulposus cells (NPCs), which are essential components of the intervertebral disc degeneration (IDD) process. In this study, IDD was treated with *Eucommia ulmoides* Oliver (Du Zhong (DZ)), and among all the active compounds, KAE was identified as the major active compound of DZ, protecting NPCs from IL-1β-induced damage through promoting cell viability, inhibiting cell senescence and apoptosis, increasing extracellular matrix (ECM) production, and decreasing ECM degradation. The mitogen-activated protein kinase (MAPK) signaling pathway may be involved, but further studies are needed to assess its role, and for the most part, it would be necessary to also assess this activity in an animal model of IDD (Table 1) [63].

#### 4.1.3. Apigenin

Apigenin is a natural flavon usually extracted from the plant *Matricaria recutita* L. (chamomile), a member of the Asteraceae (daisy) family. A study by Sang et al. reported that apigenin is involved in senescence-associated β-galactosidase activity. Firstly, the anti-oxidant activity in vitro was studied with 2,2-diphenyl-1-picrylhydrazyl (DPPH) and 2,2′-azino-bis(3-ethylbenzothiazoline-6-sulfonic acid (ABTS+) scavenging assays, which demonstrated apigenin’s strong effect. Subsequently, D-galactose was administered in an in vivo model of aging mice in order to study the eventual protective effect of apigenin. The results indicated that apigenin supplementation significantly improved aging-related modifications such as behavioral impairment, a decreased organic index, histopathological injury, and increased senescence-associated β-galactosidase (SA-β-gal) activity and the advanced glycation end product (AGE) level. Western blot analysis showed that apigenin increased Nrf2 nuclear translocation both in aging and normal young mice, with the latter presenting higher expression of Nrf2 and Nrf2 downstream gene targets, highlighting that apigenin may exert an anti-senescent effect process via activating the Nrf2 pathway [64]. Another study focused on the role of apigenin in colorectal cancer cells, also examining cell senescence in this case via in situ detection of β-galactosidase activity. The results indicated that apigenin reduced cell growth and senescence through the induction of apoptosis [65]. Other studies reported the activity of apigenin on the SASP. In particular, one study conducted on bleomycin-induced senescent BJ fibroblast by Lim et al. already described this for KAE [60], where the activity of apigenin on the SASP was confirmed on three human fibroblast strains induced to senescence by ionizing radiation, constitutive mitogen-activated protein kinase (MAPK) signaling, oncogenic RAS, or replicative exhaustion. Regarding the mechanism of action apigenin, it was found to be able to suppress SASPs following the suppression of IL-1α signaling through IRAK1, IRAK4, p38-MAPK, and NF-κB. Moreover, it was particularly potent in inhibition of the expression and secretion of chemokine interferon-γ inducible protein CXCL10 (IP10), a newly identified SASP factor [66]. Recently, Li et al. studied the effects of apigenin on a hydrogen peroxide (H_2_O_2_)- or doxorubicin (DOXO)-induced senescence model in WI-38 human embryonic lung fibroblast cells to determine the potential anti-aging effects of apigenin in vitro and its associated molecular mechanisms. The results confirmed that apigenin reduced senescence-associated β-galactosidase (SA-β-gal) activity, but they also highlighted an increased activation ratio of silent information regulator 1 (SIRT1), nicotinamide adenine dinucleotide (NAD+), and NAD+/NADH and inhibited cluster of differentiation 38 (CD38) activity in a concentration-dependent manner. Indeed, SIRT1 inhibition by SIRT1 siRNA abolished the anti-aging effect of apigenin, while cluster of differentiation 38 (CD38) inhibition by CD38 siRNA or apigenin increased the SIRT1 level and reduced H_2_O_2_-induced senescence (Table 1) [67].

#### 4.1.4. Genistein

Genistein is a naturally occurring polyphenolic flavonoid compound which structurally belongs to the class of isoflavones commonly found in various dietary vegetables, such as soybeans and fava beans. Its role in cellular senescence is supported by numerous studies. In 2016, Lee et al. reported that genistein-dependent autophagy reduced vascular smooth muscle cell (VSMC) senescence through an LKB1-AMPK-dependent mechanism [68]. Zhang et al. studied the effect of genistein on human umbilical vein endothelial cells (HUVECs) pretreated with genistein (1 µM) and then exposing the cells to ox-LDL for 12 h. Genistein was able to inhibit the ox-LDL-induced senescence, reducing the levels of p16 and p21 proteins and the activity of SA-β-gal. Moreover, it was also found to induce apoptosis, increasing LC3-II and decreasing the levels of p62, p-mTOR, and p-P70S6K, and the SIRT1/LKB1/AMPK pathway was also found to be implicated. As a consequence, genistein was indicated as a promising therapeutic to contrast senescence in HUVEC cells via inducing apoptosis [69]. A few years later, the same group demonstrated that the effect of genistein’s ox-LDL-induced mitochondrial oxidative stress and senescence occurred thanks to its action on the SIRT1–p66shc–Foxo3a pathways [70]. Using the same cell line, namely HUVECs, Guihua et al. demonstrated that genistein reduced cell senescence in the HUVEC model by suppressing the TXNIP/NLRP3 axis. The HUVECs in this case were treated with different concentrations of H_2_O_2_, which inhibited proliferation, induced cell cycle progression arrests in the G1 phase, and boosted cell senescence [71]. Recently, genistein was tested for the senescence of bone marrow mesenchymal stem cells (BMMSCs). In particular, this study was conducted on ovariectomized (OVX) rodent BMMSCs (OVX-BMMSCs). The OVX-BMMSCs showed premature senescence, elevated reactive oxygen species (ROS) levels, and mitochondria dysfunction. The treatment with genistein led to an anti-senescence effect by restoring mitochondrial homeostasis via the ERRα target. Moreover, in vivo, genistein inhibited trabecular bone loss and p16INK4a expression and upregulated sirtuin 3 (SIRT3) and peroxisome proliferator-activated receptor gamma coactivator one alpha (PGC1α) expression in the trabecular bone area of the proximal tibia in OVX rats [72]. It is worth mentioning two other studies conducted in 2019 which showed that the effects exerted by genistein are dose-dependent. Indeed, they both demonstrated that when using physiological concentrations of genistein (<2 µM), cell proliferation was obtained, while pharmacological concentrations (higher) suppressed cell proliferation. Indeed, the pharmacological concentration of genistein led to the induction of cell senescence and elevation of the ROS level (Table 1) [73,74].

#### 4.1.5. Pterostilbene

Pterostilbene is a polyphenol and an analog of resveratrol belonging to the stilbenoid family which is naturally found in grapes and berries. The role played by pterostilbene in senescence has been extensively reported in the literature [75]. Teng et al. demonstrated the mechanism by which pterostilbene managed to act on oxidative stress, inflammation, and aging in keratinocytes (HaCaT cells) exposed to particulate matter. Pterostilbene was already reported in the literature to scavenge UVB-induced ROS and protect the skin against photodamage through the Nrf2 pathway. However, the molecular mechanism was not elucidated [76,77]. They demonstrated that pterostilbene reduces the expression of inflammation and aging proteins such as aquaporin-3 (AQP-3), which is involved in skin dryness and aging [78]. Pterostilbene, together with its nicotinate analog, was tested for vascular endothelial cell senescence, where they both showed the ability to reduce cell senescence acting on β-galactosidase, downregulating p21 and p53, increasing the production of nitric oxide (NO), and increasing the activity of SIRT1 [79]. Interestingly, another study regarded the infection of human cytomegalovirus (HCMV) and HMCV-induced senescence. Among other results, pterostilbene was able to suppress HMCV-induced senescence, reducing senescence-associated β-galactosidase activity, the expression of p16, p21, and p53 proteins and also decreasing ROS [80]. One study by Jiang et al. showed that pterostilbene alleviated ethanol-triggered hepatocyte damage and senescence. In particular, it reduced the protein abundance of cellular communication network factor 1 (CCN1) in ethanol-exposed hepatocytes, which is fundamental for pterostilbene to execute its anti-senescent function. Moreover, the same group also performed in vivo studies on hepatocytes of alcohol-intoxicated mice, where pterostilbene was demonstrated to relieve the senescence-associated secretory phenotype (SASP), redox imbalance, and steatosis by suppressing hepatic CCN1 expression (Table 1) [81].

#### 4.1.6. Oleuropein Aglycone and Hydroxytyrosol

Oleuropein aglycone and hydroxytyrosol are polyphenolic compounds which are especially present in olive oil, of which they are the most abundant and studied ones. They are both reported extensively in the literature, with in vivo and in vitro studies on their anti-inflammatory activity and their role in the aging process [82]. However, only in 2017, it was demonstrated in cultured pre-senescent human lung fibroblasts (MRC5), a well-known model of cellular senescence, and in neonatal human dermal fibroblasts (NHDFs) that they both act as SASP inhibitors [83]. This was subsequently supported by another study conducted on γ-irradiated neonatal human dermal fibroblasts (NHDFs). Oleuropein aglycone and hydroxytyrosol showed a protective effect on 8 Gy irradiation-induced senescence, preserving lamin B1 expression and reducing the cGAS/STING/NFκB-mediated SASP [84]. Other studies in different cell models demonstrated the activity of both compounds and particularly that of hydroxytyrosol as an SASP inhibitor [84]. Jeon et al. in 2018 studied the anti-aging effects of hydroxytyrosol on human dermal fibroblasts (HDFs) instead, also assessing its activity as an SA-β-galactosidase inhibitor (Table 1) [85].

#### 4.1.7. Rutin

Rutin is a flavonol-type polyphenol constituent found in several plants [86] composed of the flavonol quercetin and the disaccharide rutinose, a phytochemical constituent found in a number of plants. It is a lipophilic agent characterized by relatively limited natural stability and bioavailability, mainly due to its low solubility in water, which limits the development of rutin-derived drugs [87]. Indeed, recent studies focused on new delivery approaches, such as nanomaterials, in order to improve rutin bioavailability [88]. Also, the detailed mechanism of rutin in the human body after consumption remains largely unclear. Although rutin has been known for its antitumor and antimicrobial actions, which are mainly associated with its antioxidant and anti-inflammatory activities, its potentiality in conditions involving antiaging properties and its bioactivity in the development of senotherapeutics, more specifically senomorphics, is still largely underexplored. A rather recent work by Liu et al. [89] studied the functional role of rutin as a novel senomorphic agent in targeting senescent cells and revealed the role of rutin in the early stage of SASP development and the interaction of ataxia-telangiectasia mutated (ATM) kinase with two of its direct targets: HIF1α (a master regulator of cellular and systemic homeostasis activated during senescence) and TRAF6 (part of a key signaling axis supporting the development of the SASP). This study showed rutin as an emerging natural senomorphic agent and demonstrated its potential therapeutic avenue for age-related pathologies, including cancer (Table 1).

#### 4.1.8. Luteolin

Luteolin is a natural polyphenol found in a variety of plants such as celery, sweet bell pepper, and chrysanthemums [90]. Luteolin presents antioxidant, anti-inflammatory, antitumoral, and antiapoptotic activities, which have been fully reported in the literature, supporting its protective role in neurodegenerative diseases. Interestingly, in plants, it displays protective actions against UV radiation by partially absorbing UVA and UVB radiation. As a consequence, luteolin has been studied in keratinocytes and fibroblasts as well as several immune cells in order to assess its ability to decrease adverse photobiological effects in the skin by acting as a first line of defense. Gendrisch et al. in 2021 reported that luteolin can inhibit proinflammatory mediators (e.g., IL-1β, IL-6, IL-8, IL-17, IL-22, TNF-α, and COX-2) and regulate various signaling pathways (e.g., the NF-κB, JAK–STAT and TLR signaling pathways), modulating many inflammatory processes in the skin. These data may suggest that luteolin may be a promising target to prevent or reduce photoaging effects [90]. This senomorphic activity was subsequently confirmed in 2024 by Younis et al., who demonstrated for the first time that luteolin mitigates D-gal-induced cognitive decline and hippocampal senescence, modulates oxidative stress, mitochondrial dysfunction, neuro-inflammation, and neuronal apoptosis, and promotes hippocampal neuro-regeneration. These benefits are potentially related to the upregulation of SIRT1 induced by luteolin in the hippocampus, with subsequent modulation of the glyoxalase 1 (GLO1)/AGE/RAGE signaling pathway [91]. Luteolin combined with palmitoylethanolamide is currently in clinical trials for the treatment of frontotemporal dementia (NCT04489017) (Table 1) [92].

#### 4.1.9. Hesperidin and Hesperetin

Hesperidin and hesperetin are both citrus flavonoids possessing a wide variety of reported biological effects. Hesperidin can be found abundantly in citrus fruits, as well as hesperetin, which can be viewed as a metabolite of hesperidin. It showed SASP inhibitory activity through antioxidant and anti-inflammatory effects which inhibit the production of proinflammatory cytokines [93,94,95]. Their senomorphic effect was also achieved by modulating such signaling pathways as the Nrf2, NF-κΒ, and forkhead box transcription factor (FOXO) pathways and increasing antioxidant enzyme activity [94,96]. More specifically, the effect on SASP inhibition of hesperidin was detected in human senescent chondrocytes by increasing the cellular antioxidant capacity and decreasing proinflammatory cytokines which constitute the SASP [94]. It also showed protective activity against bone loss by reducing bone resorption and NF-κB activity, and in vivo, it increased the bone mineral density in male senescent rats [93,96]. Hesperetin showed an interesting role in rheumatoid arthritis, reducing immune inflammation of the joints and modulating cytokine production and c-Jun N-terminal kinase (JNK) activity in synovial fibroblasts [97]. Despite these demonstrated and confirmed SASP effects, clinical trials involving hesperidin and hesperetin still have yet to be planned (Table 1).

#### 4.1.10. Naringenin

Naringenin is a 4′,5,7-trihydroxy flavanone, a yellow crystalline powder which shows anti-inflammatory, antioxidant, antiproliferative, anti-dyslipidaemic, and antidiabetic activity [98,99]. Naringenin was also found to modulate redox imbalance and mitochondrial metabolic activity in a model of senescent myocardial cells, showing that relevant cellular senescence markers, such as X-gal staining, cell cycle regulator levels, and the percentage of cell cycle-arrested cells, were reduced following treatment with naringenin [100]. Moreover, naringenin also displayed beneficial effects in the neural stem cell senescence model in vivo and in vitro [101]. One of the most interesting studies for our review concerns the work of Lim K. H. and Kim G. R. [102], where they demonstrated that naringenin was able to influence SASPs upregulating SIRT1 activity by acting on the NF-κB pathway and downregulating the gene expression of several inflammatory mediators and factors which produce ROS in human dermal fibroblasts, suggesting regenerative and anti-aging effects on the dermal cell structure. In 2020, Lim at al. reported a study in which naringenin was demonstrated to not suppress SASP production in bleomycin-induced senescence [60]. However, this might be due to the model used and, more importantly, variation in the mechanism which induced senescence, which may have had an impact on the composition of SASP factors [92]. Clearly, more studies are needed in order to fully understand and characterize the senomorphic potential of naringenin (Table 1).

**Table 1:** Summary of discussed senomorphic polyphenols and their mechanisms of action.

Senomorphic Polyphenol	
Resveratrol	Activates SIRT1, an NAD+-dependent deacetylase, leading to reduced cellular senescence [49]. At low concentrations, it acts as an antioxidant and prevents senescence, suppressing SASPs, while at higher concentrations, it triggers senescence or apoptosis [50].
Kaempferol	Inhibits SASP production by downregulating NF-κB signaling through the IRAK1/IκBα pathway [60]. Also enhances mitochondrial function and reduces oxidative stress [61].
Apigenin	Suppresses SASP by modulating IL-1α signaling and inhibits NF-κB and p38-MAPK pathways [66]. It activates Nrf2, reducing oxidative damage and promoting anti-senescent effects [64].
Genistein	Reduces senescence via the SIRT1/LKB1/AMPK pathway and modulates autophagy in vascular cells, reducing oxidative stress and senescence markers [69].
Pterostilbene	Modulates oxidative stress and inflammation through Nrf2 activation [76,77]. Reduces SASP factors [81] and inhibits cellular senescence in various models by increasing SIRT1 activity and reducing p21 and p53 expression [79].
Oleuropein aglycone and hydroxytyrosol	Acts as an SASP inhibitor, reducing pro-inflammatory cytokines, maintaining lamin B1 expression in irradiated cells, and protecting against radiation-induced senescence [84].
Rutin	A potent senomorphic agent targeting ATM kinase, HIF1α, and TRAF6 to inhibit SASP development. Shows potential for age-related pathologies by modulating early SASP signaling pathways [89].
Luteolin	Activates SIRT1, modulating the GLO1/AGE/RAGE pathway to reduce oxidative stress and inflammation. It also has protective effects against cognitive decline and senescence in neural cells [91].
Hesperidin and hesperetin	Inhibits SASP by modulating NF-κB, Nrf2, and FOXO pathways. Shows antioxidant and anti-inflammatory effects, protecting against cellular senescence in human chondrocytes and improving bone density in aging models [94].
Naringenin	Upregulates SIRT1, downregulates NF-κB, and modulates oxidative stress and inflammation to suppress SASP formation. Promotes regenerative and anti-aging effects in skin and myocardial cells [60].

### 4.2. Senolytic Activity

#### 4.2.1. Fisetin

Fisetin is a plant flavonol from the flavonoid group of polyphenols. It was recently reported in the literature for its senolytic activity both in vitro and in vivo. In vitro, its activity was tested in different cell models, namely in a radiation-induced senescence cell model (HUVECs) and on oxidative stress-induced senescent MEFs and genotoxin-induced senescent human fibroblasts. Fisetin leads to apoptotic cell death. An in vivo Ercc1/Δ mouse model of accelerated aging as well as a wild-type old mouse model were both treated with fisetin, obtaining reduced cellular senescence in multiple tissues and improved tissue homeostasis, alleviated age-related pathologies, and extended median and maximum lifespans, respectively. However, in the same study, fisetin also showed senomorphic activity, affecting SASP marker and senescence-associated β-galactosidase activity. The results were so promising that fisetin has entered clinical trials for chronic kidney disease (NCT03325322), skeletal health (NCT04313634), osteoarthritis (NCT04210986), COVID-19 (NCT04476953, NCT04537299, and NCT04771611), survivors of childhood cancers (NCT04733534), and frailty (NCT03675724) (Table 2).

Additionally, fisetin exhibits senomorphic activity by modulating SASP markers and reducing senescence-associated β-galactosidase activity. Thus, far only four clinical trials on fisetin’s effects on humans have been published. In men with Gulf War syndrome (NCT02909686), supplementation with 200–800 mg/day of fisetin showed no significant effects on symptom severity, fatigue, or pain. However, outcomes related to senescence or chronic inflammation were not assessed. In colorectal cancer patients, chemotherapy combined with a low dose of fisetin (100 mg/day) reduced IL-8 levels, though no effects were observed on CRP, IL-10, MMP-7, or MMP-9 levels (Farsad-Naeimi et al., 2018) [103]. In acute ischemic stroke patients, supplementation of recombinant tissue plasminogen activator with fisetin (100 mg/day) improved disease outcomes and reduced MMP-2, MMP-9, and CRP levels compared with a placebo [104]. In healthy individuals (*n* = 10) self-dosing 100 mg/day, the serum levels of SASP factors (MMP-3, MMP-9, PDGF-AA, IL-6, IL-8, MCP-1, GDF11, and GDF15) decreased, as did that of senescent peripheral blood mononuclear cells (PBMCs). However, no significant changes were found in senescent T cell levels [105].

These findings indicate fisetin’s potential to reduce inflammation and SASP markers across various conditions. Nonetheless, further studies using the “hit and run” approach with higher doses are necessary to confirm whether its effects are primarily mediated by senescent cell clearance.

Currently, fisetin has entered trials for chronic kidney disease (NCT03325322), skeletal health (NCT04313634), osteoarthritis (NCT04210986), COVID-19 (NCT04476953, NCT04537299, and NCT04771611), survivors of childhood cancers (NCT04733534), and frailty (NCT03675724) [106].

#### 4.2.2. Epigallocatechin Gallate

Epigallocatechin gallate (EGCG) is a polyphenol catechin, and it is the major component of green tea. Firstly, it is largely reported in studies in the literature in which it is addressed as a senomorphic drug, particularly for its implications in phosphoinositide 3 kinase (PI3K)/Akt/mTOR, AMPK, and sirtuin signaling, for the inhibition of ROS, iNOS, Cox-2, NF-κB, and p53 mediated cell cycle inhibition, and for being an SASP and SA-β-gal inhibitor in in vivo and in vitro models [107,108,109]. Recently, a study by Kumar et al., encouraged by these promising results, investigated whether EGCG could possibly also act as a senolytic. This research was conducted on 3T3-L1 preadipocytes cells, and EGCG (50 µM) was found to promote apoptosis in senescence cells modulating pro- and anti-apoptotic pathways, specifically Bax and Bcl-2 proteins. The author, however, pointed out that the EGCG concentrations used were higher than those generally obtained after oral or intraperitoneal treatments due to the known low bioavailability of catechins [110], and thus further studies are needed to attest to the effective relevance of this study (Table 2) [108].

#### 4.2.3. Quercetin and Dasatinib

This drug combination (quercetin + dasatinib) is the first senolytic to be discovered and reported in the literature. Quercetin is a naturally occurring flavonoid, while dasatinib is an antitumoral, a tyrosine kinase inhibitor acting on cell proliferation, migration, and apoptosis. Their combination was found to improve many age-related diseases, targeting SCAPs more efficiently than each single drug alone. It is worth mentioning that quercetin + dasatinib has already been tested in clinical trials for different pathologies, such as idiopathic pulmonary fibrosis (NCT02874989), chronic kidney disease (NCT02848131), skeletal health (NCT04313634), hematopoietic stem cell transplant survivors (NCT02652052), and Alzheimer’s disease (NCT04063124) [50,111]. The senolytic activity of this combination was due to double senolytic activity in different pathways. Indeed, dasatinib can act as a senolytic by interfering with the ephrin-dependent suppression of apoptosis and inhibition of tyrosine kinases, while quercetin can act as a senolytic by inhibiting PI3K/AKT, BCL2/BCL2L1, and TP53/P21/serpine SCAPs [112]. However, quercetin was first studied alone in 2015 in a work by Liam et al., who already reported the demonstration of its activity on SASP on a bleomycin-induced senescent BJ fibroblast model for KAE and apigenin, which appeared as a consequence of senomorphic behavior [60]. Quite recently, in 2024, Meiners et al. reported computational repurposing identification of natural compounds found in the diet to use as substitutes for dasatinib to be co-administered with quercetin based on their similarity in terms of gene expression effects. Indeed, the gene expression changes underlying the repositioning highlight apoptosis-related genes and pathways, and piperlongumine was the highest-ranking compound identified as a suitable candidate to be combined with quercetin, emulating the role of dasatinib (Table 2) [113].

#### 4.2.4. Procyanidin C1 (PCC1)

Procyanidin C1 (PCC1), a polyphenolic component especially present in grape seed extract, was reported as a novel phytochemical senotherapeutic with superior specificity and efficiency for a wider range of SC types and senescence inducers, being able to improve the health spans and lifespans of mice through its action on SCs. Notably, it is characterized by a double action, depending on the concentrations considered. Indeed, at low concentrations it presents a senomorphic activity since it inhibited SASP formation, whereas at higher concentrations it selectively kills senescent cells, possibly by increasing the production of ROS and mitochondrial dysfunction, behaving, as a consequence, as a senolytic drug. This particular, senotherapeutic activity could, at least to some extent, be due to PCC1 ability to downregulate proinflammatory gene expression and promoting ROS and mitochondrial-dependent apoptosis induction [114] (Table 2).

#### 4.2.5. Wogonin and GL-V9

Wogonin is a flavonoid found in different plants, especially in the roots of *Scutellaria baicalensis* Georgi, located mainly in Asia and Europe, presenting known antioxidant, anti-inflammatory, antiproliferative, and antimicrobial beneficial effects. Wogonin was demonstrated to reduce the SASP and downregulate the NF-κB pathway by Liam et al. in a bleomycin-induced senescent BJ fibroblast model, together with KAE, apigenin, and quercetin, showing its senomorphic way of action [60]. Because of this interesting potential therapeutic behavior and possible application in aging-related pathologies, wogonin was further studied to improve its poor bioavailability. Indeed, as with many other polyphenols, it is rapidly metabolized and excreted in urine and from intestine. Among all the analogs synthesized, 5-hydroxy-8-methoxy-2-phenyl-7-(4-(pyrrolidin-1-yl) butoxy)-4-H-chromen-4-one (GL-V9) was the most promising one [115,116]. Interestingly, GL-V9 turned out to be a senolytic, as reported in the study by Yang et al., which showed how GL-V9 eliminates senescent mouse embryonic fibroblasts (MEFs) and drug-induced senescent breast cancer cells. Moreover, it is also able to induce apoptosis in senescent MDA-MB-231 cells. Its mechanism of action concerns the alkalinization of lysosomes and the increase in mitochondria as well as reactive oxygen species (ROS). GL-V9 was also tested in vivo, where it maintained its senolytic effects, as observed in epirubicin-treated mammary tumors in MMTV-PyMT mice (Table 2) [117].

#### 4.2.6. Curcumin and Analogs

Curcumin is a polyphenol compound derived from the rhizome of the plant *Curcuma longa* (turmeric), a native Asian plant with a variety of traditional uses which belongs to the ginger family [118]. Curcumin has been extensively reported to inhibit the activity of a variety of signaling enzymes in cells which contribute to cellular survival and proliferation. There are plenty of studies describing its senolytic and senomorphic activity against senescence. Indeed, it was found to dose-dependently reduce the D-galactose-induced cardiomyocite senescence, inducing autophagy via the SIRT1/AMPK/mTOR pathway [119]. Another study conducted on canine bone marrow-derived mesenchymal stem cells (cBMSCs) highlighted the important role of curamin-induced autophagy and its effects on ameliorating cBMSC senescence (such the inhibition of SA-β-gal activities and mRNA expression of the senescence-related markers and pro-inflammatory molecules) [120]. It was also studied in vivo in aged mice, where it was shown to be effective in supplementation to prevent hepatic cellular senescent [121], but another study also showed that curcumin was able to protect the thymus against D-gal-induced senescence in ICR mice [122]. A study in 2021 concerned the use of electrospun nanofibers to realize a dual-stage release of curcumin, with the aim to increase the attachment, viability, and proliferation of adipose-derived stem cells (hADSCs), delaying cellular senescence [123]. Despite this remarkable and promising therapeutic potential, curcumin is poorly bioavailable, and for this reason, several analogs were synthesized, and compound EF24 was identified as the most promising one [124]. EF24, which presents greater bioavalibility, is an efficient broad-spectrum senolytic agent able to induce apoptosis in senescent cells by decreasing the anti-apoptotic protein family proteins, such as Bcl-2 and Bcl-XL [125], and through the enhancement of phosphatase and tensin homolog (PTEN) expression in DU145 cells [126]. Recently, its effective therapeutic potential for the treatment of idiopathic pulmonary fibrosis due to its ability to inhibit the senescence of alveolar epithelial cells responsible for driving the progression of the disease was highlighted [127]. Another curcumin analog, bis-dimethoxy curcumin, was found to protect WI38 fibroblasts from oxidative stress-induced senescence [128] (Table 2).

#### 4.2.7. Piperlongumine and Analogs

Piperlongumine is a polyphenol isolated from long pepper (*Piper longum* P.) which exerts antiplatelet, antimicrobial, antiangiogenetic, antidiabetic, antidepressant, antiatherosclerotic, neuroprotective, and anticancer properties [129]. The senolytic activity of piperlongumine has already been extensively reported in the literature, and it has been associated with the involvement of oxidation resistance 1 (OXR1) [130]. Moreover, an in vivo study regarding the acute toxicity of piperlongumine showed that even at high doses, there were no discernible clinical indications of harm. An initial series of piperlongumine analogs was synthesized in 2018 while focusing on investigation on the lactam ring, aiming to understand the SAR governing the senolytic potential of PL derivatives [124]. The same group in 2024 proposed a new series of compounds, suggesting that the reactivity of the Michael acceptor in the lactam ring is positively correlated with the senolytic effect. They identified compound 24, which displayed a remarkable 50 fold enhancement in senolytic activity compared with piperlongumine. These SAR studies are remarkably important and lead the way for the synthesis and discovery of new senotherapeutic compounds (Table 2) [131].

**Table 2:** Summary of discussed senolytic polyphenols and their mechanisms of action.

Senolytic Polyphenol	
Fisetin	Induces apoptosis in senescent cells by inhibiting survival pathways and reducing SASP markers. Shown to improve tissue homeostasis and extend lifespans in animal models [132]. In clinical trials on humans, fisetin exhibited senomorphic activity by modulating SASP markers and reducing senescence-associated β-galactosidase activity [106].
Epigallocatechin gallate (EGCG)	Modulates PI3K/Akt/mTOR signaling and promotes senescent cell apoptosis by regulating pro- and anti-apoptotic factors like Bax and Bcl-2. Shows as both senomorphic and senolytic behavior, depending on concentration and cell type [107,108,109].
Quercetin + dasatinib	This combination targets anti-apoptotic pathways (SCAPs) in senescent cells, promoting apoptosis through inhibition of PI3K/AKT and BCL2/BCL2L1 pathways. Effective at reducing senescent cells in various tissues [50,111].
Procyanidin C1 (PCC1)	Shows dual behavior; at low concentrations, it is senomorphic, while at higher concentrations, it becomes senolytic by inducing mitochondrial dysfunction and ROS generation, leading to apoptosis in senescent cells [114].
Wogonin and GL-V9	Reduces the SASP by inhibiting the NF-κB pathway and enhances mitochondrial dysfunction and ROS production in senescent cells [60]. Its derivative GL-V9 also exhibits senolytic effects [115,116].
Curcumin and analogs	Modulates SIRT1/AMPK/mTOR pathways, showing both senomorphic and senolytic effects, depending on the dose. It inhibits the SASP and enhances autophagy, reducing senescence and promoting apoptosis in higher concentrations [119].
Piperlongumine	Promotes oxidative stress in senescent cells by targeting OXR1, leading to apoptosis [130].

## 5. The Effects of Combined Phytochemicals

The consumption of vegetables, fruits, grains, and legumes, which are rich in phytochemicals, has been shown to have potential beneficial effects, particularly in the prevention and management of various disorders associated with oxidative stress, such as aging, inflammation, obesity, coronary diseases, and cancer [133].

Numerous studies have demonstrated that the effects of bioactive compounds from food, when administered as isolated dietary supplements, are not comparable to the benefits observed from diets rich in fruits, vegetables, or legumes. Researchers have proposed that phytochemicals and other bioactive compounds, when consumed in their whole food form, interact to form complexes or work synergistically, thereby exerting an enhanced effect [134].

Over the past decade, many antioxidant interactions, such as additive, synergistic, and antagonistic effects, have been identified through the combination of diverse bioactive compounds, including purified compounds (such as vitamins and phytochemicals), crude extracts, enzymes, and synthesized antioxidants [135]. The combinations of bioactive compounds and also a specific food matrix could also result in changes in the bioavailability of the compounds [136]. With regard to senescence specifically, many studies in the literature reported concerns over the use of single phytochemicals (as previously reported in this review), but extracts have also been studied, and they were revealed to be highly effective. Just to mention some of the most recent findings, the extract of Voghera peppers demonstrated a protective effect on the senescence of old, normal human diploid fibroblasts [137], and *Gingko biloba* extract was proven to effectively alleviate subchronic arsenic exposure-induced senescence of hepatocytes, acting through the inhibition of inflammation and oxidative damage in rats [138].

As a consequence, one of the key priorities for future research should be the definition of specific synergistic combinations of phytochemicals as innovative preventive nutritional strategies for the effective and safe management of aging and age-related disorders [139], as well as for promoting healthier daily dietary practices [140].

## 6. Conclusions

Cellular senescence, a critical process in aging and age-related diseases (ARDs), accumulates in tissues, driving chronic inflammation, metabolic dysregulation, and functional decline, as senescent cells produce a pro-inflammatory secretory profile (SASP) which exacerbates tissue damage and ARD progression. In this context, targeting senescence through senotherapeutics has emerged as a promising strategy to mitigate these detrimental effects and promote healthy aging [7,8]. In this review, we discussed the senotherapeutic potential of polyphenols, a varied class of dietary compounds which have shown considerable promise in preclinical studies.

Phytocompounds such as quercetin, fisetin, and luteolin have demonstrated senolytic or senomorphic activity, depending on the context, and have been shown to modulate several signaling pathways involved in cellular senescence. These compounds not only reduce the accumulation of SCs but also attenuate the release of the SASP, thereby potentially improving tissue homeostasis and reducing systemic inflammation during aging. However, while preclinical data suggest that polyphenols could be powerful agents in the fight against cellular senescence, significant challenges remain before these compounds can be translated into clinical practice.

A major limitation is the low bioavailability of polyphenolic compounds due to poor solubility, limited permeability, and rapid metabolism into inactive forms such as glucuronides and sulfates in the liver and intestines [92]. Another important consideration is the multi-target nature of polyphenols, which raises concerns about potential adverse effects and drug interactions. Specifically, polyphenols are generally regarded as antioxidants, but they may also exhibit pro-oxidant activity under certain conditions, particularly at high doses. Determining the optimal dosage ranges which maximize therapeutic benefits while minimizing potential risks is crucial [141].

Moreover, polyphenols effects on cellular senescence are often studied in isolation, but in vivo, cells are exposed to multiple forms of stress simultaneously. Therefore, to ensure the relevance of preclinical findings, future research should involve experimental models which more closely mimic the physiological conditions under which senescence occurs, like stress models that better reflect the complex microenvironment of aging tissues. Overall, there is still a need for large-scale, randomized clinical trials to validate the efficacy of polyphenols and other phytochemicals as senotherapeutics in humans [142]. In particular, clinical trials should focus on the ability of flavonoids to improve specific age-related conditions, such as idiopathic pulmonary fibrosis (IPF), osteoarthritis, and neurodegenerative diseases, where senescent cells and SASPs play a significant pathogenic role [143,144].

Another possible approach for the further study of senotherapeutics may involve personalized approaches including individual genetic variability in the metabolism of polyphenols. Genetic predispositions can significantly affect the bioavailability and efficacy of flavonoids, leading to inter-individual differences in therapeutic outcomes [145].

In conclusion, flavonoids represent a promising class of senotherapeutic agents which could play a significant role in promoting healthy aging and preventing age-related diseases. However, challenges related to bioavailability, safety, and inter-individual variability must be addressed to fully realize their potential. Future research should prioritize the real bioavailable number of polyphenols, the identification of specific senescent cell types and SASP components targeted by this class of compounds, and the validation of their efficacy in large-scale human studies. Through these efforts, polyphenols may ultimately emerge as valuable tools in the quest for healthy aging and geroprotection.

## Data Availability

No new data were created or analyzed in this study. Data sharing is not applicable to this article.

## References

[1] Hayflick L. (1965). The limited in vitro lifetime of human diploid cell strains. Exp. Cell Res..

[2] Hayflick L., Moorhead P.S. (1961). The serial cultivation of human diploid cell strains. Exp. Cell Res..

[3] Cristofalo V.J., Lorenzini A., Allen R.G., Torres C., Tresini M. (2004). Replicative senescence: A critical review. Mech. Ageing Dev..

[4] López-Otín C., Blasco M.A., Partridge L., Serrano M., Kroemer G. (2013). The hallmarks of aging. Cell.

[5] Kuilman T., Michaloglou C., Mooi W.J., Peeper D.S. (2010). The essence of senescence. Genes Dev..

[6] Liguori I., Russo G., Curcio F., Bulli G., Aran L., DELLA-Morte D., Gargiulo G., Testa G., Cacciatore F., Bonaduce D. (2018). Oxidative stress, aging, and diseases. Clin. Interv. Aging.

[7] McHugh D., Gil J. (2018). Senescence and aging: Causes, consequences, and therapeutic avenues. J. Cell Biol..

[8] Yuan L., Alexander P.B., Wang X.-F. (2020). Cellular senescence: From anti-cancer weapon to anti-aging target. Sci. China Life Sci..

[9] Demaria M., Ohtani N., Youssef S.A., Rodier F., Toussaint W., Mitchell J.R., Laberge R.-M., Vijg J., Van Steeg H., Dollé M.E. (2014). An essential role for senescent cells in optimal wound healing through secretion of PDGF-AA. Dev. Cell.

[10] Muñoz-Espín D., Cañamero M., Maraver A., Gómez-López G., Contreras J., Murillo-Cuesta S., Rodríguez-Baeza A., Varela-Nieto I., Ruberte J., Collado M. (2013). Programmed Cell Senescence during Mammalian Embryonic Development. Cell.

[11] Idda M.L., McClusky W.G., Lodde V., Munk R., Abdelmohsen K., Rossi M., Gorospe M. (2020). Survey of senescent cell markers with age in human tissues. Aging.

[12] Yousefzadeh M.J., Zhao J., Bukata C., Wade E.A., McGowan S.J., Angelini L.A., Bank M.P., Gurkar A.U., McGuckian C.A., Calubag M.F. (2020). Tissue specificity of senescent cell accumulation during physiologic and accelerated aging of mice. Aging Cell.

[13] Shree T.J., Poompavai S., Begum SM F.M., Gowrisree V., Hemalatha S., Sieni E., Sundararajan R. (2019). Cancer-Fighting Phyto-chemicals: Another Look. J. Nanomed. Biother. Discov..

[14] Ferrario G., Baron G., Gado F., Della Vedova L., Bombardelli E., Carini M., D’amato A., Aldini G., Altomare A. (2022). Polyphenols from Thinned Young Apples: HPLC-HRMS Profile and Evaluation of Their Anti-Oxidant and Anti-Inflammatory Activities by Proteomic Studies. Antioxidants.

[15] Cory H., Passarelli S., Szeto J., Tamez M., Mattei J. (2018). The Role of Polyphenols in Human Health and Food Systems: A Mini-Review. Front. Nutr..

[16] Queen B.L., Tollefsbol T.O. (2010). Polyphenols and aging. Curr. Aging Sci..

[17] Lagoumtzi S.M., Chondrogianni N. (2021). Senolytics and senomorphics: Natural and synthetic therapeutics in the treatment of aging and chronic diseases. Free Radic Biol. Med..

[18] Acosta J.C., O’Loghlen A., Banito A., Guijarro M.V., Augert A., Raguz S., Fumagalli M., Da Costa M., Brown C., Popov N. (2008). Chemokine signaling via the CXCR2 receptor reinforces senescence. Cell.

[19] Kuilman T., Michaloglou C., Vredeveld L.C., Douma S., van Doorn R., Desmet C.J., Aarden L.A., Mooi W.J., Peeper D.S. (2008). Oncogene-Induced Senescence Relayed by an Interleukin-Dependent Inflammatory Network. Cell.

[20] Birch J., Gil J. (2020). Senescence and the SASP: Many therapeutic avenues. Genes Dev..

[21] Narita M., Nuñez S., Heard E., Lin A.W., Hearn S.A., Spector D.L., Hannon G.J., Lowe S.W. (2003). Rb-mediated heterochromatin formation and silencing of E2F target genes during cellular senescence. Cell.

[22] Kim E.-C., Kim J.-R. (2019). Senotherapeutics: Emerging strategy for healthy aging and age-related disease. BMB Rep..

[23] Rajendran P., Alzahrani A.M., Hanieh H.N., Kumar S.A., Ben Ammar R., Rengarajan T., Alhoot M.A. (2019). Autophagy and senescence: A new insight in selected human diseases. J. Cell. Physiol..

[24] Fitzwalter B.E., Towers C.G., Sullivan K.D., Andrysik Z., Hoh M., Ludwig M., O’Prey J., Ryan K.M., Espinosa J.M., Morgan M.J. (2018). Autophagy Inhibition Mediates Apoptosis Sensitization in Cancer Therapy by Relieving FOXO3a Turnover. Dev. Cell.

[25] Al Bitar S., Gali-Muhtasib H. (2019). The Role of the Cyclin Dependent Kinase Inhibitor p21cip1/waf1 in Targeting Cancer: Molecular Mechanisms and Novel Therapeutics. Cancers.

[26] Sharpless N.E., Sherr C.J. (2015). Forging a signature of in vivo senescence. Nat. Rev. Cancer.

[27] Rayess H., Wang M.B., Srivatsan E.S. (2012). Cellular senescence and tumor suppressor gene p16. Int. J. Cancer.

[28] Indovina P., Marcelli E., Casini N., Rizzo V., Giordano A. (2013). Emerging roles of RB family: New defense mechanisms against tumor progression. J. Cell. Physiol..

[29] Helmbold H., Deppert W., Bohn W. (2006). Regulation of cellular senescence by Rb2/p130. Oncogene.

[30] Galanos P., Vougas K., Walter D., Polyzos A., Maya-Mendoza A., Haagensen E.J., Kokkalis A., Roumelioti F.-M., Gagos S., Tzetis M. (2016). Chronic p53-independent p21 expression causes genomic instability by deregulating replication licensing. Nat. Cell Biol..

[31] Lapasset L., Milhavet O., Prieur A., Besnard E., Babled A., Aït-Hamou N., Leschik J., Pellestor F., Ramirez J.-M., De Vos J. (2011). Rejuvenating senescent and centenarian human cells by reprogramming through the pluripotent state. Genes Dev..

[32] Childs B.G., Durik M., Baker D.J., van Deursen J.M. (2015). Cellular senescence in aging and age-related disease: From mechanisms to therapy. Nat. Med..

[33] Kirkland J.L., Tchkonia T. (2020). Senolytic drugs: From discovery to translation. J. Intern. Med..

[34] Zhu Y.I., Tchkonia T., Pirtskhalava T., Gower A.C., Ding H., Giorgadze N., Palmer A.K., Ikeno Y., Hubbard G.B., Lenburg M. (2015). The Achilles’ heel of senescent cells: From transcriptome to senolytic drugs. Aging Cell.

[35] Wang E. (1995). Senescent human fibroblasts resist programmed cell death, and failure to suppress bcl2 is involved. Cancer Res..

[36] Kudlova N., De Sanctis J.B., Hajduch M. (2022). Cellular Senescence: Molecular Targets, Biomarkers, and Senolytic Drugs. Int. J. Mol. Sci..

[37] Muñoz-Espín D., Rovira M., Galiana I., Giménez C., Lozano-Torres B., Paez-Ribes M., Llanos S., Chaib S., Muñoz-Martín M., Ucero A.C. (2018). A versatile drug delivery system targeting senescent cells. EMBO Mol. Med..

[38] Guerrero A., Guiho R., Herranz N., Uren A., Withers D.J., Martínez-Barbera J.P., Tietze L.F., Gil J. (2020). Galactose-modified duocarmycin prodrugs as senolytics. Aging Cell.

[39] Prata L.G.L., Ovsyannikova I.G., Tchkonia T., Kirkland J.L. (2018). Senescent cell clearance by the immune system: Emerging therapeutic opportunities. Semin. Immunol..

[40] Xu M., Tchkonia T., Ding H., Ogrodnik M., Lubbers E.R., Pirtskhalava T., White T.A., Johnson K.O., Stout M.B., Mezera V. (2015). JAK inhibition alleviates the cellular senescence-associated secretory phenotype and frailty in old age. Proc. Natl. Acad. Sci. USA.

[41] Tilstra J.S., Robinson A.R., Wang J., Gregg S.Q., Clauson C.L., Reay D.P., Nasto L.A., Croix C.M.S., Usas A., Vo N. (2012). NF-κB inhibition delays DNA damage–induced senescence and aging in mice. J. Clin. Investig..

[42] Lamming D.W., Ye L., Sabatini D.M., Baur J.A. (2013). Rapalogs and mTOR inhibitors as anti-aging therapeutics. J. Clin. Investig..

[43] Moiseeva O., Deschênes-Simard X., St-Germain E., Igelmann S., Huot G., Cadar A.E., Bourdeau V., Pollak M.N., Ferbeyre G. (2013). Metformin inhibits the senescence-associated secretory phenotype by interfering with IKK/NF-κB activation. Aging Cell.

[44] Naqvi K., Jabbour E., Skinner J., Anderson K., Dellasala S., Yilmaz M., Ferrajoli A., Bose P., Thompson P., Alvarado Y. (2020). Long-term follow-up of lower dose dasatinib (50 mg daily) as frontline therapy in newly diagnosed chronic-phase chronic myeloid leukemia. Cancer.

[45] Ottmann O., Saglio G., Apperley J.F., Arthur C., Bullorsky E., Charbonnier A., Dipersio J.F., Kantarjian H., Khoury H.J., Kim D.-W. (2018). Long-term efficacy and safety of dasatinib in patients with chronic myeloid leukemia in accelerated phase who are resistant to or intolerant of imatinib. Blood Cancer J..

[46] Neff F., Flores-Dominguez D., Ryan D.P., Horsch M., Schröder S., Adler T., Afonso L.C., Aguilar-Pimentel J.A., Becker L., Garrett L. (2013). Rapamycin extends murine lifespan but has limited effects on aging. J. Clin. Investig..

[47] Pang S.H.M., D’rozario J., Mendonca S., Bhuvan T., Payne N.L., Zheng D., Hisana A., Wallis G., Barugahare A., Powell D. (2021). Mesenchymal stromal cell apoptosis is required for their therapeutic function. Nat. Commun..

[48] Tripathi U., Misra A., Tchkonia T., Kirkland J.L. (2021). Impact of Senescent Cell Subtypes on Tissue Dysfunction and Repair: Importance and Research Questions. Mech. Ageing Dev..

[49] Chen C., Zhou M., Ge Y., Wang X. (2020). SIRT1 and aging related signaling pathways. Mech. Ageing Dev..

[50] Zhang L., Pitcher L.E., Prahalad V., Niedernhofer L.J., Robbins P.D. (2023). Targeting cellular senescence with senotherapeutics: Senolytics and senomorphics. FEBS J..

[51] Xia L., Wang X.X., Hu X.S., Guo X.G., Shang Y.P., Chen H.J., Zeng C.L., Zhang F.R., Chen J.Z. (2008). Resveratrol reduces endothelial progenitor cells senescence through augmentation of telomerase activity by Akt-dependent mechanisms. Br. J. Pharmacol..

[52] Csiszar A., Sosnowska D., Wang M., Lakatta E.G., Sonntag W.E., Ungvari Z. (2012). Age-associated proinflammatory secretory phenotype in vascular smooth muscle cells from the non-human primate macaca mulatta: Reversal by resveratrol treatment. J. Gerontol. A Biol. Sci. Med. Sci..

[53] Giovannelli L., Pitozzi V., Jacomelli M., Mulinacci N., Laurenzana A., Dolara P., Mocali A. (2011). Protective Effects of Resveratrol Against Senescence-Associated Changes in Cultured Human Fibroblasts. J. Gerontol. Ser. A.

[54] Baur J.A., Pearson K.J., Price N.L., Jamieson H.A., Lerin C., Kalra A., Prabhu V.V., Allard J.S., Lopez-Lluch G., Lewis K. (2006). Resveratrol improves health and survival of mice on a high-calorie diet. Nature.

[55] Miller R.A., Harrison D.E., Astle C.M., Baur J.A., Boyd A.R., de Cabo R., Fernandez E., Flurkey K., Javors M.A., Nelson J.F. (2011). Rapamycin, But Not Resveratrol or Simvastatin, Extends Life Span of Genetically Heterogeneous Mice. J. Gerontol. Biol. Sci. Med. Sci..

[56] Strong R., Miller R.A., Astle C.M., Baur J.A., de Cabo R., Fernandez E., Guo W., Javors M., Kirkland J.L., Nelson J.F. (2013). Evaluation of Resveratrol, Green Tea Extract, Curcumin, Oxaloacetic Acid, and Medium-Chain Triglyceride Oil on Life Span of Genetically Heterogeneous Mice. J. Gerontol. A Biol. Sci. Med. Sci..

[57] Liu J., Jiao K., Zhou Q., Yang J., Yang K., Hu C., Zhou M., Li Z. (2021). Resveratrol Alleviates 27-Hydroxycholesterol-Induced Senescence in Nerve Cells and Affects Zebrafish Locomotor Behavior via Activation of SIRT1-Mediated STAT3 Signaling. Oxidative Med. Cell. Longev..

[58] Ali D., Chen L., Kowal J.M., Okla M., Manikandan M., AlShehri M., AlMana Y., AlObaidan R., AlOtaibi N., Hamam R. (2020). Resveratrol inhibits adipocyte differentiation and cellular senescence of human bone marrow stromal stem cells. Bone.

[59] Shaito A., Posadino A.M., Younes N., Hasan H., Halabi S., Alhababi D., Al-Mohannadi A., Abdel-Rahman W.M., Eid A.H., Nasrallah G.K. (2020). Potential Adverse Effects of Resveratrol: A Literature Review. Int. J. Mol. Sci..

[60] Lim H., Park H., Kim H.P. (2015). Effects of flavonoids on senescence-associated secretory phenotype formation from bleomycin-induced senescence in BJ fibroblasts. Biochem. Pharmacol..

[61] Yao X., Jiang H., Li Y., Gao Q., Xu Y.N., Kim N. (2019). Kaempferol alleviates the reduction of developmental competence during aging of porcine oocytes. Anim. Sci. J..

[62] Kim J., Kim H.-S., Choi D.-H., Choi J., Cho S.Y., Kim S.-H., Baek H.-S., Yoon K.D., Son S.W., Son E.D. (2022). Kaempferol tetrasaccharides restore skin atrophy via PDK1 inhibition in human skin cells and tissues: Bench and clinical studies. Biomed. Pharmacother..

[63] Wang X., Tan Y., Liu F., Wang J., Liu F., Zhang Q., Li J. (2023). Pharmacological network analysis of the functions and mechanism of kaempferol from Du Zhong in intervertebral disc degeneration (IDD). J. Orthop. Transl..

[64] Sang Y., Zhang F., Wang H., Yao J., Chen R., Zhou Z., Yang K., Xie Y., Wan T., Ding H. (2017). Apigenin exhibits protective effects in a mouse model ofd-galactose-induced aging via activating the Nrf2 pathway. Food Funct..

[65] Zohreh B., Masoumeh V., Fakhraddin N., Omrani G.H.R. (2019). Apigenin-mediated Alterations in Viability and Senescence of SW480 Colorectal Cancer Cells Persist in The Presence of L-thyroxine. Anti-Cancer Agents Med. Chem..

[66] Perrott K.M., Wiley C.D., Desprez P.-Y., Campisi J. (2017). Apigenin suppresses the senescence-associated secretory phenotype and paracrine effects on breast cancer cells. GeroScience.

[67] Li B.S., Zhu R.Z., Lim S.-H., Seo J.H., Choi B.-M. (2021). Apigenin Alleviates Oxidative Stress-Induced Cellular Senescence via Modulation of the SIRT1-NAD+-CD38 Axis. Am. J. Chin. Med..

[68] Lee K.Y., Kim J.-R., Choi H.C. (2016). Genistein-induced LKB1–AMPK activation inhibits senescence of VSMC through autophagy induction. Vasc. Pharmacol..

[69] Zhang H., Yang X., Pang X., Zhao Z., Yu H., Zhou H. (2019). Genistein protects against ox-LDL-induced senescence through enhancing SIRT1/LKB1/AMPK-mediated autophagy flux in HUVECs. Mol. Cell. Biochem..

[70] Zhang H., Pang X., Yu H., Zhou H. (2022). Genistein suppresses ox-LDL-elicited oxidative stress and senescence in HUVECs through the SIRT1-p66shc-Foxo3a pathways. J. Biochem. Mol. Toxicol..

[71] Wu G., Li S., Qu G., Hua J., Zong J., Li X., Xu F. (2021). Genistein alleviates H_2_O_2_ -induced senescence of human umbilical vein endothelial cells via regulating the TXNIP/NLRP3 axis. Pharm. Biol..

[72] Li M., Yu Y., Xue K., Li J., Son G., Wang J., Qian W., Wang S., Zheng J., Yang C. (2023). Genistein mitigates senescence of bone marrow mesenchymal stem cells via ERRα-mediated mitochondrial biogenesis and mitophagy in ovariectomized rats. Redox Biol..

[73] Weis K.E., Raetzman L.T. (2019). Genistein inhibits proliferation and induces senescence in neonatal mouse pituitary gland explant cultures. Toxicology.

[74] Jenie R.I., Amalina N.D., Ilmawati G.P.N., Utomo R.Y., Ikawati M., Khumaira A., Kato J.Y., Meiyanto E. (2019). Cell Cycle Modulation of CHO-K1 Cells Under Genistein Treatment Correlates with Cells Senescence, Apoptosis and ROS Level but in a Dose-Dependent Manner. Adv. Pharm. Bull..

[75] Li Y., Li S., Lin C. (2018). Effect of resveratrol and pterostilbene on aging and longevity. BioFactors.

[76] Li H., Jiang N., Liang B., Liu Q., Zhang E., Peng L., Deng H., Li R., Li Z., Zhu H. (2017). Pterostilbene protects against UVB-induced photo-damage through a phosphatidylinositol-3-kinase-dependent Nrf2/ARE pathway in human keratinocytes. Redox Rep..

[77] Wang B.-J., Chiu H.-W., Lee Y.-L., Li C.-Y., Wang Y.-J., Lee Y.-H. (2018). Pterostilbene Attenuates Hexavalent Chromium-Induced Allergic Contact Dermatitis by Preventing Cell Apoptosis and Inhibiting IL-1β-Related NLRP3 Inflammasome Activation. J. Clin. Med..

[78] Teng W.-L., Huang P.-H., Wang H.-C., Tseng C.-H., Yen F.-L. (2021). Pterostilbene Attenuates Particulate Matter-Induced Oxidative Stress, Inflammation and Aging in Keratinocytes. Antioxidants.

[79] Zhang L., Zheng J., Tie X., Lin T., Yang W., Li Z., Zou Y., Guan G., Liu P., Luo W. (2021). Pterostilbene and its nicotinate derivative ameliorated vascular endothelial senescence and elicited endothelium-dependent relaxations via activation of sirtuin 1. Can. J. Physiol. Pharmacol..

[80] Wang S., Zhou X., He X., Ma S., Sun C., Zhang J., Xu X., Jin W., Yan J., Lin P. (2022). Suppressive effects of pterostilbene on human cytomegalovirus (HCMV) infection and HCMV-induced cellular senescence. Virol. J..

[81] Jiang Y., Zhou Y., Xu W., Wang X., Jin H., Bao X., Lu C. (2023). Induction of Sestrin2 by pterostilbene suppresses ethanol-triggered hepatocyte senescence by degrading CCN1 via p62-dependent selective autophagy. Cell Biol. Toxicol..

[82] Giovannelli L. (2012). Beneficial effects of olive oil phenols on the aging process: Experimental evidence and possible mechanisms of action. Nutr. Aging.

[83] Menicacci B., Cipriani C., Margheri F., Mocali A., Giovannelli L. (2017). Modulation of the Senescence-Associated Inflammatory Phenotype in Human Fibroblasts by Olive Phenols. Int. J. Mol. Sci..

[84] Frediani E., Scavone F., Laurenzana A., Chillà A., Tortora K., Cimmino I., Leri M., Bucciantini M., Mangoni M., Fibbi G. (2022). Olive phenols preserve lamin B1 expression reducing cGAS/STING/NFκB-mediated SASP in ionizing radiation-induced senescence. J. Cell. Mol. Med..

[85] Jeon S., Choi M. (2018). Anti-inflammatory and anti-aging effects of hydroxytyrosol on human dermal fibroblasts (HDFs). Biomed. Dermatol..

[86] Budzynska B., Faggio C., Kruk-Slomka M., Samec D., Nabavi S.F., Sureda A., Devi K.P., Nabavi S.M. (2019). Rutin as Neuroprotective Agent: From Bench to Bedside. Curr. Med. Chem..

[87] Gullón B., Lú-Chau T.A., Moreira M.T., Lema J.M., Eibes G. (2017). Rutin: A review on extraction, identification and purification methods, biological activities and approaches to enhance its bioavailability. Trends Food Sci. Technol..

[88] Negahdari R., Bohlouli S., Sharifi S., Dizaj S.M., Saadat Y.R., Khezri K., Jafari S., Ahmadian E., Jahandizi N.G., Raeesi S. (2021). Therapeutic benefits of rutin and its nanoformulations. Phytother. Res..

[89] Liu H., Xu Q., Wufuer H., Li Z., Sun R., Jiang Z., Dou X., Fu Q., Campisi J., Sun Y. (2024). Rutin is a potent senomorphic agent to target senescent cells and can improve chemotherapeutic efficacy. Aging Cell.

[90] Gendrisch F., Esser P.R., Schempp C.M., Wölfle U. (2021). Luteolin as a modulator of skin aging and inflammation. BioFactors.

[91] Younis R.L., El-Gohary R.M., Ghalwash A.A., Hegab I.I., Ghabrial M.M., Aboshanady A.M., Mostafa R.A., El-Azeem A.H.A., Farghal E.E., Belal A.A. (2024). Luteolin Mitigates D-Galactose-Induced Brain Ageing in Rats: SIRT1-Mediated Neuroprotection. Neurochem. Res..

[92] Mbara K.C., Devnarain N., Owira P.M.O. (2022). Potential Role of Polyphenolic Flavonoids as Senotherapeutic Agents in Degenerative Diseases and Geroprotection. Pharm. Med..

[93] Habauzit V., Sacco S.M., Gil-Izquierdo A., Trzeciakiewicz A., Morand C., Barron D., Pinaud S., Offord E., Horcajada M.-N. (2011). Differential effects of two citrus flavanones on bone quality in senescent male rats in relation to their bioavailability and metabolism. Bone.

[94] Tsai Y.-F., Chen Y.-R., Chen J.-P., Tang Y., Yang K.-C. (2019). Effect of hesperidin on anti-inflammation and cellular antioxidant capacity in hydrogen peroxide-stimulated human articular chondrocytes. Process. Biochem..

[95] Rizza S., Muniyappa R., Iantorno M., Kim J.-A., Chen H., Pullikotil P., Senese N., Tesauro M., Lauro D., Cardillo C. (2011). Citrus Polyphenol Hesperidin Stimulates Production of Nitric Oxide in Endothelial Cells while Improving Endothelial Function and Reducing Inflammatory Markers in Patients with Metabolic Syndrome. J. Clin. Endocrinol. Metab..

[96] Fu Z., Chen Z., Xie Q., Lei H., Xiang S. (2018). Hesperidin protects against IL-1β-induced inflammation in human osteoarthritis chondrocytes. Exp. Ther. Med..

[97] Choi E.M., Lee Y.S. (2010). Effects of hesperetin on the production of inflammatory mediators in IL-1β treated human synovial cells. Cell. Immunol..

[98] Vafeiadou K., Vauzour D., Lee H.Y., Rodriguez-Mateos A., Williams R.J., Spencer J.P.E. (2009). The citrus flavanone naringenin inhibits inflammatory signalling in glial cells and protects against neuroinflammatory injury. Arch. Biochem. Biophys..

[99] Nyane N.A., Tlaila T.B., Malefane T.G., Ndwandwe D.E., Owira P.M.O. (2017). Metformin-like antidiabetic, cardio-protective and non-glycemic effects of naringenin: Molecular and pharmacological insights. Eur. J. Pharmacol..

[100] Da Pozzo E., Costa B., Cavallini C., Testai L., Martelli A., Calderone V., Martini C. (2017). The Citrus Flavanone Naringenin Protects Myocardial Cells against Age-Associated Damage. Oxidative Med. Cell. Longev..

[101] Gao J., Wu Y., He D., Zhu X., Li H., Liu H., Liu H. (2020). Anti-aging effects of Ribes meyeri anthocyanins on neural stem cells and aging mice. Aging.

[102] Lim K.H., Kim G.R. (2018). Inhibitory effect of naringenin on LPS-induced skin senescence by SIRT1 regulation in HDFs. Biomed. Dermatol..

[103] Farsad-Naeimi A., Alizadeh M., Esfahani A., Aminabad E.D. (2018). Effect of fisetin supplementation on inflammatory factors and matrix metalloproteinase enzymes in colorectal cancer patients. Food Funct..

[104] Wang L., Cao D., Wu H., Jia H., Yang C., Zhang L. (2019). Fisetin prolongs therapy window of brain ischemic stroke using tissue plasminogen activator: A double-blind randomized placebo-controlled clinical trial. Clin. Appl. Thromb. Hemost..

[105] Hambright W.S., Duke V.R., Goff A.D., Goff A.W., Minas L.T., Kloser H., Gao X., Huard C., Guo P., Lu A. (2024). Clinical validation of C12FDG as a marker associated with senescence and osteoarthritic phenotypes. Aging Cell.

[106] Tavenier J., Nehlin J.O., Houlind M.B., Rasmussen L.J., Tchkonia T., Kirkland J.L., Andersen O., Rasmussen L.J.H. (2024). Fisetin as a senotherapeutic agent: Evidence and perspectives for age-related diseases. Mech. Ageing Dev..

[107] Sharma R., Kumar R., Sharma A., Goel A., Padwad Y. (2021). Long term consumption of green tea EGCG enhances healthspan and lifespan in mice by mitigating multiple aspects of cellular senescence in mitotic and post-mitotic tissues, gut dysbiosis and immunosenescence. bioRxiv.

[108] Kumar R., Sharma A., Kumari A., Gulati A., Padwad Y., Sharma R. (2019). Epigallocatechin gallate suppresses premature senescence of preadipocytes by inhibition of PI3K/Akt/mTOR pathway and induces senescent cell death by regulation of Bax/Bcl-2 pathway. Biogerontology.

[109] Han D.-W., Lee M.H., Kim B., Lee J.J., Hyon S.-H., Park J.-C. (2012). Preventive effects of epigallocatechin-3-O-gallate against replicative senescence associated with p53 acetylation in human dermal fibroblasts. Oxidative Med. Cell. Longev..

[110] Lambert J.D., Lee M.-J., Lu H., Meng X., Hong J.J.J., Seril D.N., Yang C.S., Sturgill M.G. (2003). Epigallocatechin-3-gallate is absorbed but extensively glucuronidated following oral administration to mice. J. Nutr..

[111] Gerdes E.O.W., Zhu Y., Tchkonia T., Kirkland J.L. (2020). Discovery, development, and future application of senolytics: Theories and predictions. FEBS J..

[112] Wiley C.D., Campisi J. (2021). The metabolic roots of senescence: Mechanisms and opportunities for intervention. Nat. Metab..

[113] Meiners F., Hinz B., Boeckmann L., Secci R., Sueto S., Kuepfer L., Fuellen G., Barrantes I. (2024). Computational identification of natural senotherapeutic compounds that mimic dasatinib based on gene expression data. Sci. Rep..

[114] Xu Q., Fu Q., Li Z., Liu H., Wang Y., Lin X., He R., Zhang X., Ju Z., Campisi J. (2021). The flavonoid procyanidin C1 has senotherapeutic activity and increases lifespan in mice. Nat. Metab..

[115] Li L., Chen P., Ling Y., Song X., Lu Z., He Q., Li Z., Lu N., Guo Q. (2011). Inhibitory effects of GL-V9 on the invasion of human breast carcinoma cells by downregulating the expression and activity of matrix metalloproteinase-2/9. Eur. J. Pharm. Sci..

[116] Xing H., Ren C., Kong Y., Ni Q., Wang Z., Zhao D., Li N., Chen X., Lu Y. (2019). Determination of GL-V9, a derivative of wogonin, in rat plasma by UPLC–MS/MS and its application to a pharmacokinetic study after oral and pulmonary administration. Biomed. Chromatogr..

[117] Yang D., Tian X., Ye Y., Liang Y., Zhao J., Wu T., Lu N. (2021). Identification of GL-V9 as a novel senolytic agent against senescent breast cancer cells. Life Sci..

[118] Slika L., Patra D. (2020). Traditional Uses, Therapeutic Effects and Recent Advances of Curcumin: A Mini-Review. Mini Rev. Med. Chem..

[119] Yang L., Shi J., Wang X., Zhang R. (2022). Curcumin Alleviates D-Galactose-Induced Cardiomyocyte Senescence by Promoting Autophagy via the SIRT1/AMPK/mTOR Pathway. Evidence-Based Complement. Altern. Med..

[120] Deng J., Ouyang P., Li W., Zhong L., Gu C., Shen L., Cao S., Yin L., Ren Z., Zuo Z. (2021). Curcumin Alleviates the Senescence of Canine Bone Marrow Mesenchymal Stem Cells during In Vitro Expansion by Activating the Autophagy Pathway. Int. J. Mol. Sci..

[121] Lee D.-Y., Lee S.-J., Chandrasekaran P., Lamichhane G., O’connell J.F., Egan J.M., Kim Y. (2023). Dietary Curcumin Attenuates Hepatic Cellular Senescence by Suppressing the MAPK/NF-κB Signaling Pathway in Aged Mice. Antioxidants.

[122] Li J.-H., Wei T.-T., Guo L., Cao J.-H., Feng Y.-K., Guo S.-N., Liu G.-H., Ding Y., Chai Y.-R. (2021). Curcumin protects thymus against D-galactose-induced senescence in mice. Naunyn-Schmiedebergs Arch. Pharmacol..

[123] Serati-Nouri H., Rasoulpoor S., Pourpirali R., Sadeghi-Soureh S., Esmaeilizadeh N., Dadashpour M., Roshangar L., Zarghami N. (2021). In vitro expansion of human adipose-derived stem cells with delayed senescence through dual stage release of curcumin from mesoporous silica nanoparticles/electrospun nanofibers. Life Sci..

[124] Adams B.K., Ferstl E.M., Davis M.C., Herold M., Kurtkaya S., Camalier R.F., Hollingshead M.G., Kaur G., Sausville E.A., Rickles F.R. (2004). Synthesis and biological evaluation of novel curcumin analogs as anti-cancer and anti-angiogenesis agents. Bioorganic Med. Chem..

[125] Li W., He Y., Zhang R., Zheng G., Zhou D. (2019). The curcumin analog EF24 is a novel senolytic agent. Aging.

[126] Yang C.H., Yue J., Sims M., Pfeffer L.M. (2013). The curcumin analog EF24 targets NF-κB and miRNA-21, and has potent anticancer activity in vitro and in vivo. PLoS ONE.

[127] Zhang Y., Liu J., Zheng R., Hou K., Zhang Y., Jia T., Lu X., Samarawickrama P.N., Jia S., He Y. (2024). Curcumin analogue EF24 prevents alveolar epithelial cell senescence to ameliorate idiopathic pulmonary fibrosis via activation of PTEN. Phytomedicine.

[128] Malavolta M., Bracci M., Santarelli L., Sayeed M.A., Pierpaoli E., Giacconi R., Costarelli L., Piacenza F., Basso A., Cardelli M. (2018). Inducers of Senescence, Toxic Compounds, and Senolytics: The Multiple Faces of Nrf2-Activating Phytochemicals in Cancer Adjuvant Therapy. Mediat. Inflamm..

[129] Bezerra D.P., Pessoa C., de Moraes M.O., Saker-Neto N., Silveira E.R., Costa-Lotufo L.V. (2013). Overview of the therapeutic potential of piplartine (piperlongumine). Eur. J. Pharm. Sci..

[130] Zhang X., Zhang S., Liu X., Wang Y., Chang J., Zhang X., Mackintosh S.G., Tackett A.J., He Y., Lv D. (2018). Oxidation resistance 1 is a novel senolytic target. Aging Cell.

[131] Zhang X., He Y., Liu X., Zhang X., Shi P., Wang Y., Zhou D., Zheng G. (2024). Design and optimization of piperlongumine analogs as potent senolytics. Bioorganic Med. Chem. Lett..

[132] Yousefzadeh M.J., Zhu Y., McGowan S.J., Angelini L., Fuhrmann-Stroissnigg H., Xu M., Ling Y.Y., Melos K.I., Pirtskhalava T., Inman C.L. (2018). Fisetin is a senotherapeutic that extends health and lifespan. EBioMedicine.

[133] Sajadimajd S., Bahramsoltani R., Iranpanah A., Patra J.K., Das G., Gouda S., Rahimi R., Rezaeiamiri E., Cao H., Giampieri F. (2020). Advances on Natural Polyphenols as Anticancer Agents for Skin Cancer. Pharmacol. Res..

[134] Liu R.H. (2004). Potential Synergy of Phytochemicals in Cancer Prevention: Mechanism of Action. J. Nutr..

[135] Stinco C.M., Heredia F.J., Vicario I.M., Meléndez-Martínez A.J. (2016). In vitro antioxidant capacity of tomato products: Relationships with their lycopene, phytoene, phytofluene and alpha-tocopherol contents, evaluation of interactions and correlation with reflectance measurements. LWT Food Sci. Technol..

[136] Mishra A.K., Singh R., Rawat H., Kumar V., Jagtap C., Jain A. (2024). The influence of food matrix on the stability and bioavailability of phytochemicals: A comprehensive review. Food Humanit..

[137] De Luca F., Gola F., Azzalin A., Casali C., Gaiaschi L., Milanesi G., Vicini R., Rossi P., Bottone M.G. (2024). A Lombard Variety of Sweet Pepper Regulating Senescence and Proliferation: The Voghera Pepper. Nutrients.

[138] Chen X., Wu F., Chen C., Ren Q., Zhang A. (2024). Ginkgo Biloba Extract Can Antagonize Subchronic Arsenite Exposure-Induced Hepatocyte Senescence by Inhibiting Oxidative Damage and Inflammation in Rats. Biol. Trace Elem. Res..

[139] Abdolmaleky H.M., Zhou J.-R. (2023). Underlying Mechanisms of Brain Aging and Neurodegenerative Diseases as Potential Targets for Preventive or Therapeutic Strategies Using Phytochemicals. Nutrients.

[140] Chen X., Li H., Zhang B., Deng Z. (2022). The synergistic and antagonistic antioxidant interactions of dietary phytochemical combinations. Crit. Rev. Food Sci. Nutr..

[141] Calabrese E.J. (2021). Hormesis Mediates Acquired Resilience: Using Plant-Derived Chemicals to Enhance Health. Annu. Rev. Food Sci. Technol..

[142] Childs B.G., Gluscevic M., Baker D.J., Laberge R.-M., Marquess D., Dananberg J., van Deursen J.M. (2017). Senescent cells: An emerging target for diseases of ageing. Nat. Rev. Drug Discov..

[143] Coppé J.-P., Rodier F., Patil C.K., Freund A., Desprez P.-Y., Campisi J. (2011). Tumor suppressor and aging biomarker p16(INK4a) induces cellular senescence without the associated inflammatory secretory phenotype. J. Biol. Chem..

[144] Watanabe S., Kawamoto S., Ohtani N., Hara E. (2017). Impact of senescence-associated secretory phenotype and its potential as a therapeutic target for senescence-associated diseases. Cancer Sci..

[145] Favari C., de Alvarenga J.F.R., Sánchez-Martínez L., Tosi N., Mignogna C., Cremonini E., Manach C., Bresciani L., Del Rio D., Mena P. (2024). Factors driving the inter-individual variability in the metabolism and bioavailability of (poly)phenolic metabolites: A systematic review of human studies. Redox Biol..

